# m6A Regulator-Mediated Methylation Modification Patterns and Tumor Microenvironment Cell-Infiltration Characterization in Head and Neck Cancer

**DOI:** 10.3389/fcell.2021.803141

**Published:** 2022-02-07

**Authors:** Qinghui Yang, Feng Xu, Aiwen Jian, Hongmei Yu, Tao Ye, Weiqi Hu

**Affiliations:** ^1^ Department of Oncology, Minhang Branch, Zhongshan Hospital, Fudan University, Shanghai, China; ^2^ Key Laboratory of Whole-Period Monitoring and Precise Intervention of Digestive Cancer (SMHC), Minhang Hospital & AHS, Fudan University, Shanghai, China; ^3^ Department of Nuclear Medicine, Shanghai Ninth People’s Hospital, Shanghai Jiao Tong University School of Medicine, Shanghai, China; ^4^ School of Basic Medical Sciences, Shandong University, Jinan, China; ^5^ Department of Otolaryngology, Minhang Branch, Zhongshan Hospital, Fudan University, Shanghai, China

**Keywords:** m6A, methylation, head and neck cancer, bioinformatics, tumor microenvironment

## Abstract

**Background:** Recently, RNA modifications have emerged as essential epigenetic regulators of gene expression. However, the mechanism of how RNA *N* ^6^-methyladenosine (m6A) modification interacts with tumor microenvironment (TME) infiltration remains obscure.

**Methods:** A total of 876 head and neck cancer samples considering 21 m6A regulators were included and analyzed to determine the m6A modification patterns. These modification patterns were then correlated with TME immune cell-infiltrating characteristics. A scoring system, the m6Ascore, was constructed using principal component analysis algorithms to quantify m6A modification of tumors.

**Results:** Three m6A modification patterns were identified, with TME infiltrating characteristics highly consistent with tumors with three distinct immune phenotypes, including immune-inflamed, immune-exclude, and immune-desert phenotypes. It was demonstrated that the identification of the m6A modification patterns via m6Ascore could predict tumor progression, subtypes, TME stromal activity, variation of relevant genes, and patient prognosis. Low m6Ascore, identified to be an inflamed phenotype, is found to be associated with low stroma activity and tumor mutation burden, high survival probability, increased tumor neoantigen burden, and enhanced response to anti-PD-1/L1 immunotherapy. The therapeutic advantages and clinical benefits of patients with low m6Ascore were further verified in two immunotherapy cohorts.

**Conclusion:** This study identified the significant role that the m6A modification played in the formation of TME characteristics. A more comprehensive understanding of the m6A modification patterns and their correlation with TME infiltration will contribute to the discovery of immunotherapy strategies with better efficacy.

## Introduction

Head and neck cancer, a prevalent heterogeneous carcinoma with significant malignancy originating from the squamous cells, constitutes the sixth most common cancer worldwide, located in the mucous membrane of the oral cavity, oropharynx, paranasal sinuses, nasopharynx, larynx, and hypopharynx (Marur and Forastiere, 2016; Bray et al., 2018). The disease is known for poor prognosis and high morbidity, with almost half of the patients diagnosed with head and neck cancer failing to achieve cure and cancer relapse occurring despite intensive combined therapy. The inadequacy of efficient biomarkers results in advanced-stage diagnosis, contributing to a shorter 5-year survival rate (Lingen, 2010; Franzmann et al., 2012; Ayaz et al., 2013; Gupta et al., 2015). Major triggering factors of head and neck cancer include alcohol and tobacco consumption, and virus infection, such as human papillomavirus (HPV) and Epstein–Barr virus (Franceschi et al., 1990; Gillison et al., 2000; Sisk et al., 2003; Argiris et al., 2008). An intricate multistep process of carcinogenesis induced by the accumulation of genetic and epigenetic alterations of tumor suppressor genes, oncogenic signaling pathways, and regulation of RNA modification can be triggered by frequent exposure to these carcinogens. Emerging evidence indicated that changes in the epigenetic landscape, including DNA methylation, histone modification, noncoding RNA activity, and RNA methylation as well, play an indispensable role in the carcinogenesis of head and neck cancer (Castilho et al., 2017; Zhang et al., 2018). Among these suspected oncogenic mechanisms, RNA modifications have currently occurred as crucial epigenetic regulators of gene expression, which may contribute significantly to the comprehension of pathogenic mechanisms of head and neck cancer and the discovery of potential therapeutic treatments and biomarkers for early detection and improved prognosis.

Chemical modifications on RNA, including deamination and methylation, are currently acknowledged to have significant regulatory effects on gene expression. Internal cRNA modifications, prevalent in forms of 5-methylcytosine (m5C), *N* ^6^-methyladenosine (m6A), *N* ^1^-methyladenosine (m1A), ribose-methylation (2′-*O*-Me), and pseudo-uridine (Ψ), serve as the third layer of epigenetics in all living beings, with a total amount of more than 150 types being identified (Desrosiers et al., 1974; Perry et al., 1974; Wei et al., 1975; Boccaletto and Bagiński, 2021). Among these modifications, methylation of *N* ^6^ adenosine (m6A) on messenger RNA (mRNA) is the most common and abundant internal modification pattern found in a wide range of eukaryotes and at least 25% of all RNA (Alarcón et al., 2015; Zhao et al., 2017). Similar to DNA modifications in mammalian cells, RNA modifications typically display dynamic reversibility regulated by methyltransferases, demethylases, and binding proteins, in other words, “writers,” “erasers,” and “readers,” allowing organisms to adjust to changing environment (Yang et al., 2018). Methyltransferases, including ZC3H13, CBLL1, FMR1, KIAA1429, METTL14, RBM15, METTL3, and WTAP, catalyze the methylation of m6A, while the demethylation process is regulated by demethylases ALKBH3, ALKBH5, and FTO. The “readers” are a group of RNA-binding proteins consisting of YTHDF1-3, YTHDC1-2, LRPPRC, IGF2BP1-3, HNRNPC, HNRNPA2B1, and ELAVL1, with the high specificity of m6A motif recognition, playing a significant role in regulating the function of m6A. To date, various biological processes are identified to be associated with the m6A modification of RNA, including stem cell proliferation and differentiation, tumorigenesis, and tumor microenvironment (TME) infiltration of immune cells (Klungland et al., 2016; Cui et al., 2017). m6A methylation of RNA is also found to be involved in the activation of the immune system, *via* upregulation and downregulation of certain biological pathways. For example, in 2019, Wang et al. have identified the role of the METTL3-mediated m6A modification in dendritic cell (DC) activation and DC-based T-cell response, by causing downregulation of the downstream effector molecules of the TLR4/NK-κB pathway (Wang et al., 2019).

Rising awareness has been paid to the role that the m6A modification plays in the characterization of TME and its therapeutic potential in immunotherapy for several cancers (He and He, 2021; Wardowska, 2021; Wu et al., 2021). Liu et al. utilized prostate cancer as the subject to analyze the correlation between the m6A modification patterns and the TME characterization. It was found that the m6A modification had a significant influence on TME, and the low m6Ascore group with a poor prognosis was more responsive to immunotherapy, receiving more clinical benefits (Liu et al., 2021a). In a study by Huang et al. on hepatocellular carcinoma and a study by Liu et al. on breast cancer, however, the high m6Ascore group had more clinical advantages over the low m6Ascore group and was more responsive to immunotherapy (Liu et al., 2021b; Wu and Bai, 2021). Despite distinct effects that the m6A modification has on different cancer, it is universally affirmed that the m6A modification is significantly correlated with TME characterization and the efficacy of immunotherapy. Therefore, thorough comprehension of these m6A regulators and their interactions can be beneficial in understanding the association between the m6A modification and post-transcriptional regulation, thus contributing to the classification of head and neck cancer patients and subsequent specified therapy.

Currently, the treatment for head and neck cancer mainly involves surgical eradication, radiotherapy (RT), chemotherapy (CT), and epidermal growth factor receptor (EGFR)-targeted drug cetuximab for both HPV(+) and HPV(−) subtypes (Moskovitz et al., 2018). Unfortunately, current therapies only present a limited efficacy and high instability, due to individual heterogeneity and complexity of the disease (Alsahafi et al., 2019). Despite traditional treatments, various immunotherapeutic approaches for head and neck cancer treatment are under investigation, including immune checkpoint inhibitors (ICIs), tumor vaccines, cell-based therapies, and cytokine therapy (Ferris, 2015; Cramer et al., 2019).

Immunotherapy, such as immunological checkpoint blockade (ICB), which has been a hotspot in recent years, has exhibited inspiring potential in providing treatment with higher specification, efficacy, and durable adverse effects. Recent studies suggest that PD-1/PD-L1 blockade immunotherapy exhibited more satisfactory curative effects with fewer undesirable adverse effects compared with traditional therapies for patients with head and neck cancer in advanced stages (Ferris et al., 2016; Seiwert et al., 2016; Addeo et al., 2019; Cohen et al., 2019). Mounting evidence has elucidated that besides tumor cells, the TME also plays a considerable role in tumor progression (Chen et al., 2021). TME is a complex composed of tumor cells and stromal cells including infiltrating immune cells (effector or regulatory T cells, M1/M2 macrophages, N1/N2 neutrophils, and natural killer cells), myeloid-derived suppressor cells (MDSCs), new blood vessels, and secreted factors such as cytokines and chemokines. With advances in knowledge regarding TME diversity and characterization, it has become clearer that TME has a significant effect on the efficiency of ICB treatment. Therefore, a comprehensive understanding of the heterogeneity, diversity, and complexity of the TME landscape can contribute to the identification of different tumor immune phenotypes and the improvement of immunotherapy efficacy in head and neck cancer patients.

The reason for choosing head and neck cancer for this analysis is threefold. First of all, head and neck cancer is one of the 10th most common cancers worldwide. Secondly, it is known for poor prognosis and high morbidity, making it urgent to find efficient biomarkers or scoring systems for early discovery and prediction of the prognosis. Thirdly, the m6A modification is proved to be associated with immune activation and suppression, along with the efficacy of immunotherapy confirmed in several studies. Hence, a more comprehensive understanding of the m6A modification in head and neck cancer can contribute to personalized treatment and subsequently better clinical outcomes.

In this study, the genomic data of 876 head and neck cancer samples were selected to thoroughly analyze the m6A modification patterns and associated TME infiltration characterization. Via subsequent analyses, the samples were classified into three clusters, which were to some extent resembling the phenotype classification of the immune-inflamed tumor, immune-desert tumor, and immune-excluded tumor, suggesting a prominent influence that the m6A modification has on the formation of individual TME characteristics. Moreover, a series of scoring systems designed to quantify the m6A modification pattern in individuals were established, considering the heterogeneity of the m6A modification between individuals.

## Methods

### Head and Neck Cancer Dataset Source and Preprocessing

Public gene-expression data and full clinical annotation included in this study were extracted from Gene Expression Omnibus (GEO) and The Cancer Genome Atlas (TCGA) databases. Four eligible head and neck cancer cohorts (GSE41613, GSE42743, GSE65858, and TCGA-HNSC), with patients without survival information excluded, were collected for further analyses. To process microarray data from Affymetrix, the raw “CEL” files were downloaded, and a robust multiarray averaging method with the affy and simpleaffy packages was adopted for background adjustment and quantile normalization. The normalized matrix files for microarray data from other platforms were directly downloaded. Datasets and RNA sequencing data (FPKM value) of gene expression in TCGA-Illumina RNAseq were downloaded from the Genomic Data Commons (GDC; https://portal.gdc.cancer.gov/) using the R package TCGAbiolinks. The FPKM values were then transformed into transcripts per kilobase million (TPM) values. The “ComBat” algorithm of the sva package was used to correct batch effects from non-biological technical biases. The information of all eligible head and neck cancer datasets is exhibited in Supplementary Table S1.

### Unsupervised Clustering for 21 m6A Regulators

A total of 21 m6A regulators, composed of 7 writers (CBLL1, KIAA1429, METTL14, METTL13, RBM15, RBM15B, WTAP), 3 erasers (FTO, ALKBH5, ALKBH3), and 11 readers (YTHDF1, YTHDF2, YTHDF3, YTHDC1, YTHDC2, LRPPRC, IGF2BP2, IGF2BP3, HNRNPC, HNRNPA2B1, and ELAVL1), were detected from the GEO datasets for subsequent analyses. Unsupervised clustering analysis was used to identify different m6A modification patterns based on the expression of these regulators and categorized head and neck cancer samples into distinct clusters. The number and stability of individual clusters were determined using a consensus clustering algorithm, which was further guaranteed using the ConsensuClusterPlus package.

### Gene Set Variation Analysis and Functional Annotation

Gene Set Variation Analysis (GSVA) enrichment analysis using the “GSVA” R packages was performed to identify the difference in biological processes between distinct m6A modification patterns. An adjusted *p*-value of less than 0.05 was considered statistically significant.

### Estimation of Tumor Microenvironment Cell Infiltration

The single-sample gene set enrichment analysis (ssGSEA) algorithm was applied to quantify the relative abundance of individual cell infiltration in the head and neck cancer TME. From the study of Charoentong, the gene set for marking each immune cell type included in TME infiltration was obtained, including activated B cell, activated CD4 T cell, activated CD8 T cell, activated DC, and CD56bright natural killer cell. To demonstrate the relative abundance of each TME infiltrating cell in each sample, the enrichment scores were calculated by ssGSEA.

### Identification of Differentially Expressed Genes Between m6A Distinct Phenotypes

Head and neck cancer patients included in this study were classified into three separate m6A modification clusters based on the expression of 21 m6A regulators. The m6A-related genes and differentially expressed genes (DEGs) between individual modification patterns were determined utilizing the empirical Bayesian approach of the limma R package. An adjusted *p-*value of less than 0.001 was defined to be statistically significant.

### Generation of m6A Gene Signature and Its Correlation With Other Related Biological Processes

The m6A gene signature, termed the m6Ascore, was a set of scoring systems constructed to quantify the m6A modification patterns of individual head and neck cancer tumors. To establish this scoring system, a series of data processing were performed.

Firstly, the DEGs identified were normalized among all samples, and the overlap genes were extracted. Three gene clusters were determined using the consensus clustering algorithm, with their stability further examined. Then, a prognostic analysis for each gene in the signature was performed using a univariate Cox regression model. Genes with significant prognostic value were extracted for further analyses. Subsequent principal component analysis (PCA) was performed to construct m6A relevant gene signature, with principal components 1 and 2 selected as signature scores. The m6Ascore was defined using the following method:

$$\text{M}6\text{Ascore}=\sum \left({\text{PC}1}_{\text{i}}{+\text{PC}2}_{\text{i}}\right)$$

In the equation above, “i” represents the expression of m6A phenotype-related genes.

Afterward, a correlation analysis was performed to elucidate the correlation between m6A gene signature and other related biological processes.

### Collection of Genomic and Clinical Information of Immune Checkpoint Blockade

Public ICB gene expression profiles with complete clinical information were systematically researched, and two immunotherapeutic cohorts were eventually included, which constituted the anti-PD-1 group and anti-PD-L1 groups in this study.

### Statistical Analysis

First, Spearman’s and distance correlation analyses were used to determine the correlations coefficients between the TME infiltrating immune cells and expression of m6A regulators. The characterization of individual cohorts was inspected utilizing one-way ANOVA and Kruskal–Wallis tests. Then the patients were classified into the high and low m6Ascore groups based on the maximally selected log-rank statistics. The survival curves of distinct cohorts for the prognostic analysis were generated using the Kaplan–Meier method, with the significance of differences identified via log-rank tests. Finally, the specificity and sensitivity of m6Ascore were assessed and visualized by generating a receiver operating characteristic (ROC) curve, with the area under the curve (AUC) quantified using the pROC R package. All *p*-values with statistical meaning were two-sided, with an adjusted *p-*value of less than 0.05 regarded as statistically significant.

## Results

### Overview of Genetic Variation of m6A Regulators in Head and Neck Cancer

**FIGURE 1:**
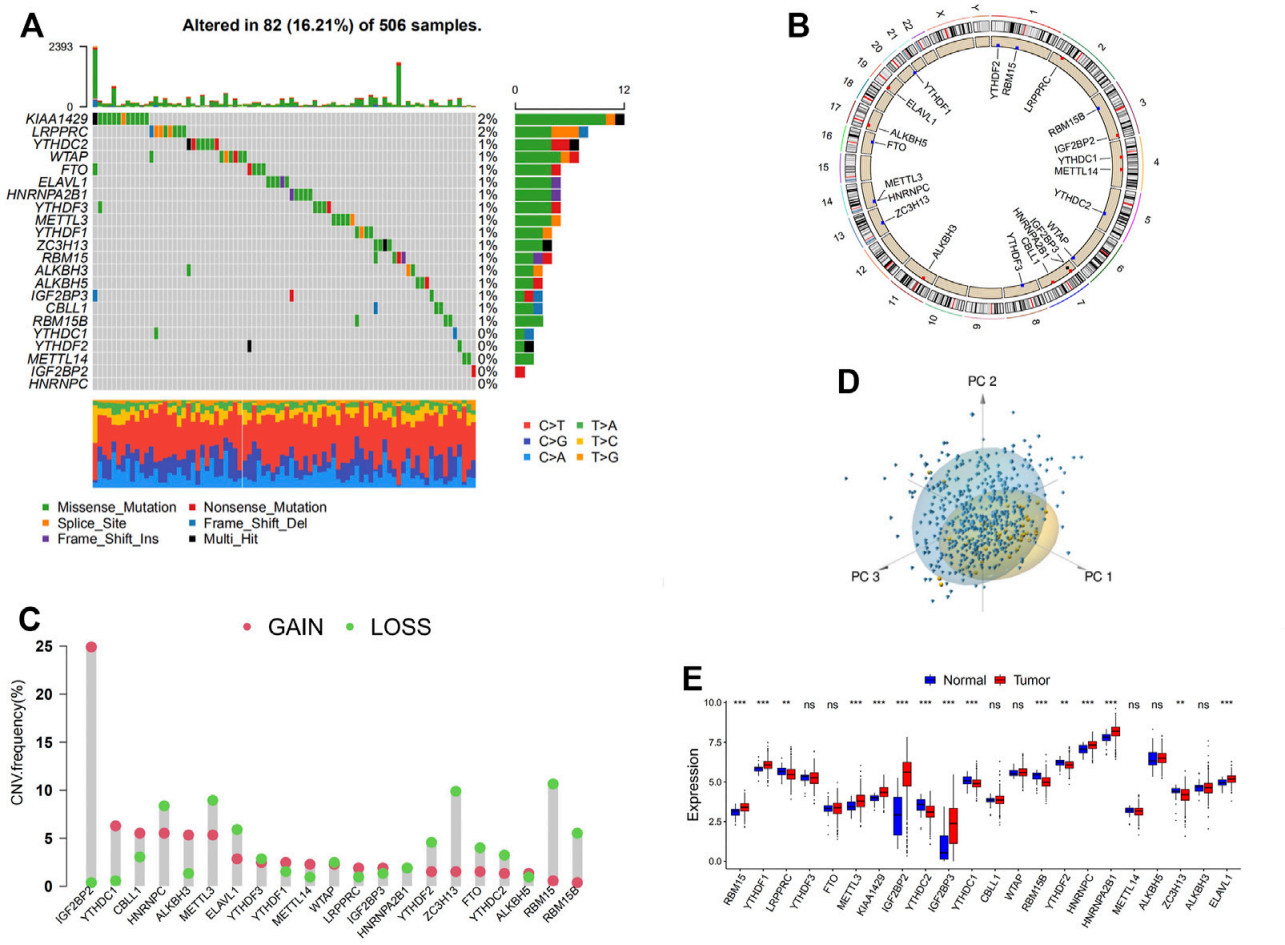
Landscape of genetic and expression variation of m6A regulators in head and neck cancer. **(A)** The mutation frequency of 21 m6A regulators in 506 patients with head and neck cancer. Each column represents individual patients. The upper barplot shows tumor mutational burden (TMB). The number on the right indicates the mutation frequency in each regulator. The right barplot shows the proportion of each variant type. The stacked barplot below shows a fraction of conversions in each sample. **(B)** The copy number variation (CNV) frequency of m6A regulators in GSE41613 cohort. The height of the column represents the alteration frequency. The deletion frequency, blue dot; the amplification frequency, red dot. **(C)** The location of CNV alteration of m6A regulators on 23 chromosomes using GSE41613 cohort. **(D)** Principal component analysis for the expression profiles of 21 m6A regulators to distinguish tumors from normal samples in GSE41613 cohort. Two subgroups without intersection were identified, indicating the tumors and normal samples were well distinguished based on the expression profiles of m6A regulators. Tumors are marked in blue, and normal samples are marked in yellow. **(E)** The expression of 21 m6A regulators between normal tissues and cancer tissues. Tumor, red; normal, blue. The upper and lower ends of the boxes represent the interquartile range of values. The lines in the boxes represent median value, and black dots show outliers. The asterisks represent the statistical *p*-value (*p < 0.05; **p < 0.01; ***p < 0.001).

In this study, 21 m6A regulators, composed of 8 writers, one eraser, and 12 readers, were eventually identified and included for further analyses. To acquire a comprehensive overview of the genetic variation of m6A regulators in head and neck cancer, the incidence of copy number variations (CNVs) and somatic mutations in the head and neck cancer samples were summarized. Out of 506 samples, 82 (16.21%) experienced mutation of m6A regulators, among which KIAA1429 presented the highest mutation frequency seconded by LRPPRC. However, no mutation was found in a percentage of readers (YTHDC1, YTHDF2, IGF2BP2, HNRNPC) and METTL14 (Figure 1A). Further analyses revealed a significant mutation co-occurrence relationship between FTO and YTHDF2, RBM15B, and YTHDF1, and also RBM15 and CBLL1 (Supplementary Figure S1A). In the investigation of the CNV alteration frequency, the majority of the regulators showed amplification in CNV, except for HNRNPC, METTL3, ELAVL1, YTHDF3, WTAP, HNRNPA2B1, YTHDF2, ZC3H13, FTO, YTHDC2, RBM15, and RBM15B, which had a widespread frequency of CNV deletion (Figure 1B). The CNV alteration of m6A regulators was located on 15 chromosomes, as shown in Figure 1C. According to the PCA conducted on the samples, as presented in Figure 1D, samples with head and neck cancer can be distinguished completely from normal samples. The mRNA expression levels of the m6A regulators in head and neck cancer were then analyzed to explore whether the genetic variations mentioned above have influences over the expression of these regulators, and the answer was positive. It was found that the CNV alteration contributed significantly to the perturbations on the m6A regulator expression. Subsequently, in the comparison between head and neck cancer samples and normal tissues, a prominently higher expression of m6A regulators, such as IGF2BP2, was found in cancer samples (Figures 1B,E). High heterogeneity between the normal and head and neck cancer samples was indicated in the alteration of the genetic and expressional landscape of m6A regulators through the above analyses. The imbalance of the expression of m6A regulators in the head and neck cancer samples was hence suggested to contribute significantly to the occurrence and progression of head and neck cancer.

### m6A Methylation Modification Patterns Mediated by 21 Regulators

**FIGURE 2:**
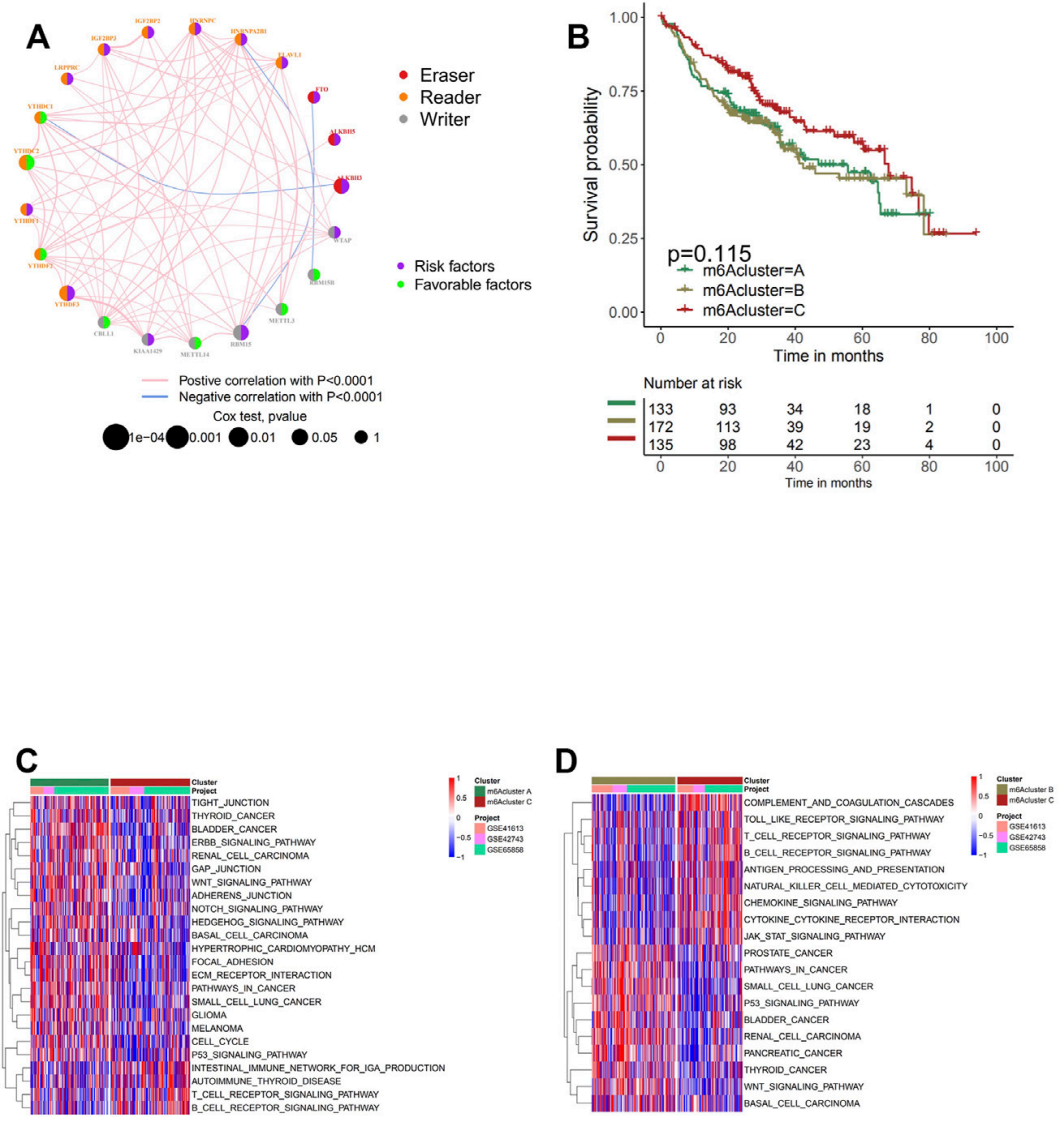
Patterns of m6A methylation modification and biological characteristics of each pattern. **(A)** The interaction between m6A regulators in head and neck cancer. The circle size represents the effect of each regulator on the prognosis, and the range of values calculated by log-rank test was *p* < 0.001, *p* < 0.01, *p* < 0.05, and *p* < 0.1. Purple in the right part of the circle, risk factors of prognosis; green in the right part of the circle, protective factors of prognosis. The lines linking regulators show their interactions, and thickness shows the correlation strength between regulators. The color in the right part of the circle represents whether the regulator is “eraser” (red), “reader” (orange), or “writer” (gray). Negative correlation is marked in blue, and positive correlation with red. **(B)** Survival analyses for the three m6A modification patterns based on 440 patients with head and neck cancer from four cohorts (GSE41613, GSE42743, GSE65858, and GSE65858PFS) including 133 cases in m6A cluster A, 172 cases in m6A cluster B, and 135 cases in m6A cluster C. Kaplan–Meier curves with log-rank *p*-value 0.115 show a significant survival difference among three m6A modification patterns. m6A cluster C shows significantly better overall survival than the other two m6A cluster. **(C, D)** Gene Set Variation Analysis (GSVA) enrichment analysis shows the activation states of biological pathways in distinct m6A modification patterns. The heatmap was used to visualize these biological processes; and red represents activated pathways, and blue represents inhibited pathways. The head and neck cohorts were used as sample annotations. **(C)** m6A cluster A vs. m6A cluster C; **(D)** m6A cluster B vs. m6A cluster C.

For subsequent analyses, three GEO datasets with OS data and clinical information available for later processing (GSE41613, GSE42743, and GSE65858) were included in one meta-cohort. The interaction and connection between each m6A regulator and the correlation between the expression of the regulator and the disease were visualized in the network Figure 2A. Positive correlations were widely found between multiple regulators especially among regulators in the reader and writer functional categories, while eraser regulators were found to be relatively independent of the rest of the regulators. A negative correlation with *p* < 0.0001 was found only between the regulator HNRNPA2B1 and RBM15B, FTO and RBM15, ALKBH3, and YTHDC1. Subsequently, the mutation co-occurrence and exclusion analyses for 21 m6A regulators were performed in the following study. It was found that the mutation occurrence of reader genes and eraser genes were largely dependent on different types of writer and eraser genes (Supplementary Figure S1A). The tumors with a mutation of certain writer genes (RBM15B, CBLL1, WTAP, KIAA1429, and RBM15) showed a high rate of mutation in reader genes (ZC3H13, YTHDF3, YTHDF1, and ZC3H13). Writer gene KIAA1429 was also found to be positively correlated with the mutation of eraser gene ALKBH3. It was noticed that tumors with a high rate of mutation in eraser genes (FTO and ALKBH3) also exhibited high expression of reader genes (YTHDF2 and YTHDC2), while the mutation of FTO was found to co-occur with the mutation of both writer gene KIAA1429 and reader gene IGF2BP3. We further investigated the correlation between writer genes and different reader and eraser genes (Supplementary Figures S2A–H). It was found that head and neck cancer samples with high expression of writer genes KIAA1429 were in positive correlation with eraser genes ALKBH3 and FTO, while tumors with a high expression of writer genes (RBM15, RBM15B, and CBLL1) exhibited a low expression of eraser genes (ALKBH3 and FTO). The high expression of WTAP, METTL14, and METTL3 did not affect the expression of eraser genes significantly. Cross-talk among the regulation of the m6A regulator genes was suggested to play a crucial role in the development of different m6A regulation patterns and TME cell-infiltrating characterization between individual tumors. Based on different expressions of 21 m6A regulators in head and neck cancer samples, the R package of ConsensusClusterPlus was utilized to categorize patients with the m6A modification patterns with a quantitative difference. Three clusters with distinct m6A modification patterns were eventually identified via unsupervised clustering, which were later termed m6A clusters A, B, and C (Supplementary Figure S2I). Although subsequent prognostic analyses to explore the clinical feature of the three clusters demonstrated no prominent survival advantage in any of the m6A clusters, we cannot yet come to the conclusion that the m6A modification exerts no influence on the prognosis of head and neck cancer, since only the m6A regulators were included in this clustering system (Figure 2B).

### Tumor Microenvironment Cell Infiltration Characteristics in Distinct m6A Modification Patterns

The biological processes of separating the m6A modification patterns of m6A clusters A, B, and C were visualized through the heatmap acquired from GSVA enrichment analysis. Clusters A and B presented significant increment in stromal and carcinogenic activation pathways, including GLIOMA, WNT signaling pathway, and pathways in cancer, while activation of the immune system was observed in cluster C. Prominent enrichment of pathways associated with complete immune activation was found in cluster C, while downregulation was shown in clusters A and B (Figures 2C,D).

**FIGURE 3:**
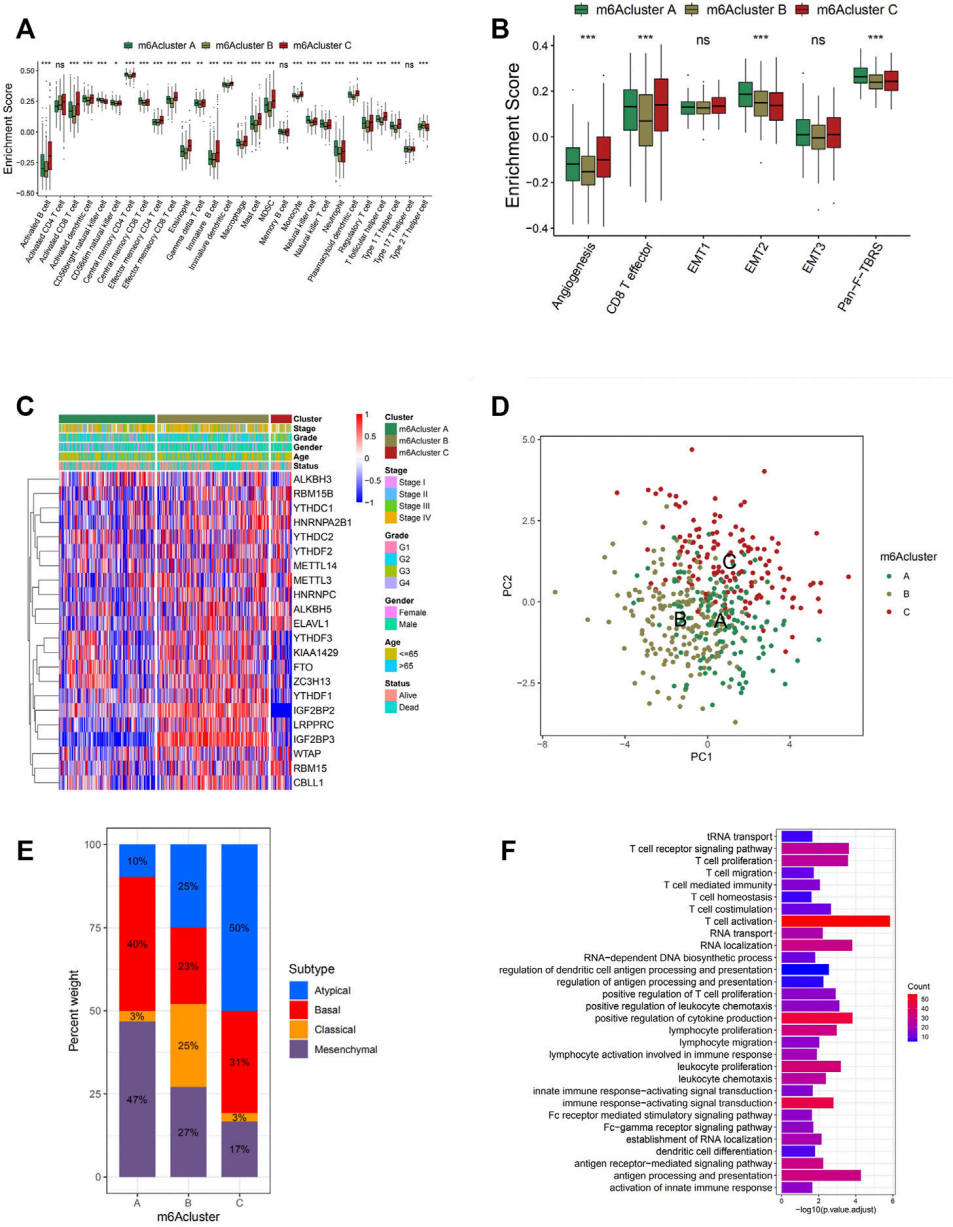
Tumor microenvironment (TME) cell infiltration characteristics and transcriptome traits in distinct m6A modification patterns. **(A)** The abundance of each TME infiltrating cell in three m6A modification patterns. The upper and lower ends of the boxes represent the interquartile range of values. The lines in the boxes represent median value, and black dots show outliers. The asterisks represent the statistical *p*-value (*p < 0.05; **p < 0.01; ***p < 0.001). **(B)** Differences in stroma-activated pathways including epithelial-to-mesenchymal transition (EMT), TGF beta, and angiogenesis pathways among three distinct m6A modification patterns. The statistical differences among the three modification patterns were tested by the one-way ANOVA test. The asterisks represent the statistical *p*-value (*p < 0.05; **p < 0.01; ***p < 0.001). **(C)** Unsupervised clustering of 21 m6A regulators in the GSE65858 cohort. The m6A cluster, molecular subtypes, tumor stage, survival status, and age were used as patient annotations. Red represents high expression of regulators, and blue represents low expression. **(D)** Principal component analysis for the transcriptome profiles of three m6A modification patterns, showing a remarkable difference in transcriptome between different modification patterns. **(E)** The proportion of four molecular subtypes of head and neck cancer in the three modification patterns. Atypical subtype, blue; basal subtype, red; classical subtype, orange; mesenchymal subtype, purple. **(F)** Functional annotation for m6A-related genes using Gene Ontology (GO) enrichment analysis. The color depth of the barplots represents the number of genes enriched.

In general, cluster C scored the highest in various types of immune cells enrichment, which was consistent with its high survival probability (Figure 3A and Figure 2B). According to the recent criterion of tumor immune phenotype classification, the three m6A clusters can be categorized into three immune phenotypes separately. The immune-inflamed tumor shows abundant immune cell infiltration both inside tumor cell parenchyma and at their surrounding stroma, which matched with the characteristics of m6A cluster C. Cluster B, which fitted the definition of tumors with immune-desert phenotype, showed minimal infiltration of immune cells in TME, especially CD8+ T cells, resulting in insensitivity and poor efficacy of ICB therapy. Surprisingly, it was noticed that considerable immune cell infiltration enrichment, including activated B cells and neutrophils, was observed in m6A cluster A, contradictory with the relatively low survival probability obtained from the previous analysis. It was reported in recent studies that besides the immune-inflamed phenotype, the immune-excluded phenotype of tumors also presented increment of immune cells but was only restrained in the stromal surrounding of tumor cells (Pai et al., 2020). Moreover, emerging evidence demonstrated an immunosuppressive effect of the activation of stroma in TME, inhibiting the normal function of T cells (Chen and Mellman, 2017). Therefore, via the information above and the results from GSVA analyses (Figure 2D), it was suspected that activation of stromal activation pathways suppressed the antitumor effect of immune cells, thus resulting in significantly lower survival probability compared with other clusters. This speculation was further confirmed by subsequent analyses, the result of which indicated prominent enrichment in stroma activity in cluster A, including the activation of EMT2, EMT3, and Pan-F-TBRS (Figure 3B). Furthermore, the specific correlation between the individual types of TME infiltration cell and m6A regulator was examined via Spearman’s correlation analyses (Supplementary Figure S3A). Several regulators, including “readers” YTHDF1, LRPPRC, ELAVL1, and IGF2BP1-3 and “eraser” ALKBH3, present a nonnegligible negative correlation with various TME infiltrating immune cells. To verify the conjecture, unsupervised clustering of 21 m6A regulators in three clusters was analyzed via data acquired from GSE41613 and GSE42743 (Figure 3C). The expression of m6A regulators was enhanced in cluster B, whereas in clusters A and C, the expression was generally decreased. The decrement was especially obvious for the expression of m6A regulator gene IGF2BP2 and IGF2BP3, which may play an essential role in the immunosuppressive response. Through PCA, it was noticed that the three m6A clusters presented distinguishing characteristics in the expression of m6A regulators (Figure 3D). Cluster A was characterized by decreased expression of METTL3 and YTHDC2; cluster B can be differentiated by enhanced expression of HNRNPA2B1 and HNRNPC; and cluster C presented prominent decrement in the expression of IGF2BP2 and IGF2BP3. Subsequent one-way ANOVA test further confirmed the significant differences that existed between three m6A clusters (Supplementary Figure S4F).

### m6A Methylation Modification Patterns in GSE65858 Cohort

To further explore the characteristics of these m6A modification phenotypes in the different clinical traits and biological behaviors, we fixed attention on the GSE65858 cohort, which comprised 130 head and neck cancer patients, and offered the most comprehensive clinical annotation. Similar to all head and neck cancer datasets clustering, unsupervised clustering also discovered three fully distinct patterns of the m6A modification in the GSE65858 cohort (Supplementary Figures S4A–D and Figures 3C,D). There was a significant distinction that existed on the m6A transcriptional profile among three different m6A modification patterns (Figure 3D). m6A cluster A was characterized by the decreased expression of IGF2BP3; m6A cluster B showed high expression of IGF2BP2 and IGF2BP3; and m6A cluster C exhibited significant decreases in the expression of IGF2BP2 and KIAA1429 (Figure 3C).

In prior researches, four statistically significant gene expression subtypes were detected in head and neck cancer, which were referred to as atypical (AT), basal (BA), classical (CL), and mesenchymal (MS) (Chung et al., 2004; Walter et al., 2013). We noted that tumors with m6A cluster A patterns presented a large proportion of MS subtypes, which was confirmed to be related to elevated expression of genes associated with the epithelial-to-mesenchymal transition (EMT). It was verified by the results mentioned above that EMT2 and Wnt signaling pathways were significantly enriched in m6A cluster A (Figure 2C and Figure 3E). This explains the poor prognosis and survival rates of m6A cluster A with a higher probability of infiltration and distant metastasis. A prognostic analysis was conducted on the GSE65858 cohort, and no significant survival difference among the three clusters was found with a *p*-value of 0.114 (Supplementary Figure S4E). However, due to only one cluster being included in this analysis and other DEGs related to the disease were not considered, it cannot be concluded that no significant survival difference existed in clusters with different m6A modification patterns.

### Generation of m6A Gene Signatures and Functional Annotation

**FIGURE 4:**
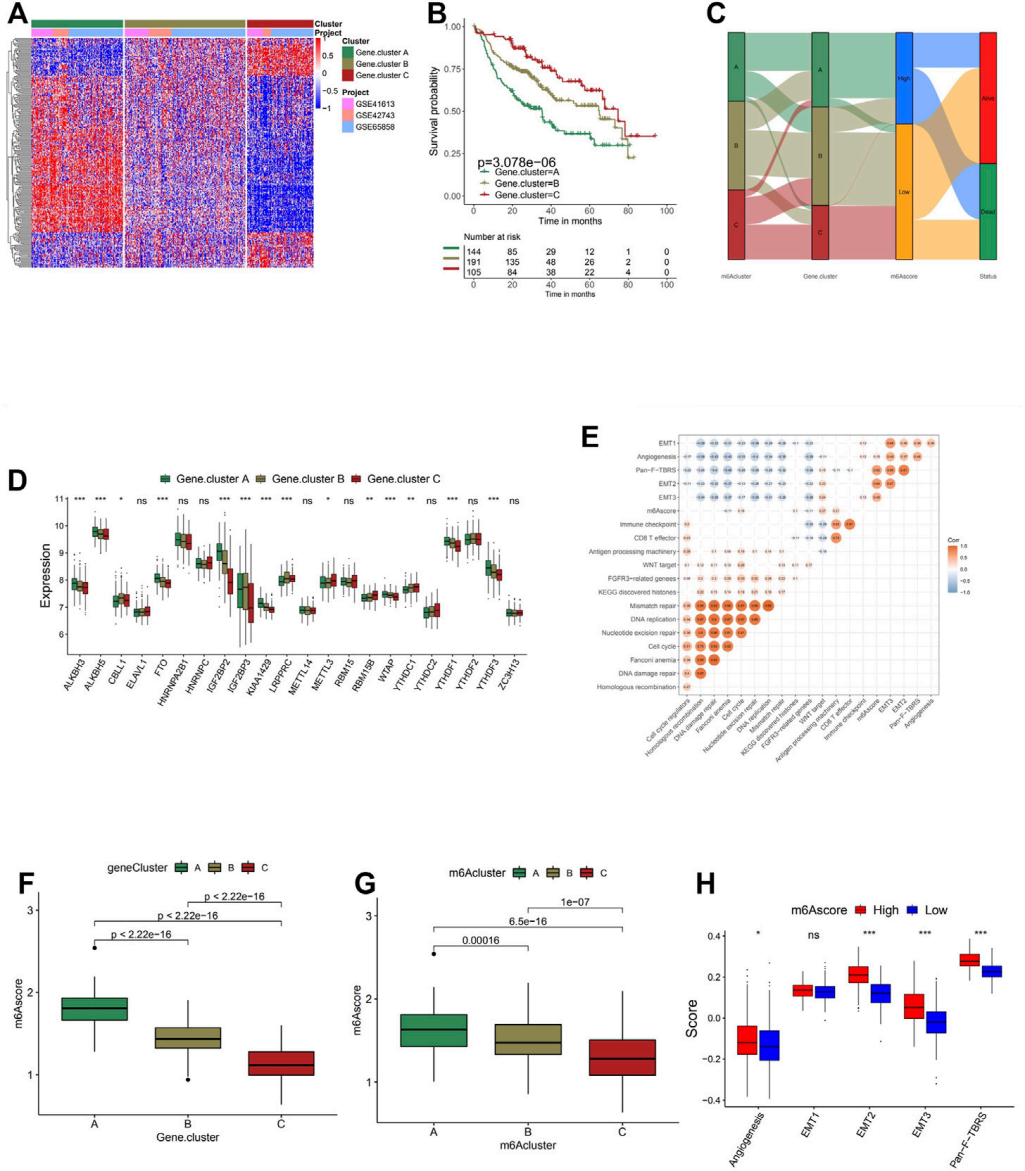
Construction of m6A signatures. **(A)** Unsupervised clustering of overlapping m6A phenotype-related genes to classify patients into different genomic subtypes, termed as m6A gene clusters A–C. The gene clusters and molecular subtypes were used as patient annotations. **(B)** Kaplan–Meier curves indicate that m6A modification genomic phenotypes were markedly related to the overall survival of 440 patients, of which 144 cases were in gene cluster A, 191 cases in gene cluster B, and 105 cases in gene cluster C (*p* < 0.0001, log-rank test). **(C)** The expression of 21 m6A regulators in three gene clusters. The upper and lower ends of the boxes represent the interquartile range of values. The lines in the boxes represent the median value, and black dots show outliers. The asterisks represent the statistical *p*-value (*p < 0.05; **p < 0.01; ***p < 0.001). The one-way ANOVA test was used to test the statistical differences among three gene clusters. **(D)** Alluvial diagram showing the changes of m6A clusters, gene cluster, m6Ascore, and outcomes. **(E)** Correlations between m6Ascore and the known gene signatures using Spearman’s analysis. Negative correlation is marked in blue and positive correlation with orange. **(F)** Differences in m6Ascore among three gene clusters. The Kruskal–Wallis test was used to compare the statistical difference between three gene clusters (*p* < 0.001). **(G)** Differences in m6Ascore among three m6A modification patterns (*p* < 0.001, Kruskal–Wallis test). **(H)** Differences in stroma-activated pathways between high m6Ascore and low m6Ascore groups. The upper and lower ends of the boxes represent the interquartile range of values. The lines in the boxes represent the median value. The asterisks represent the statistical *p*-value (*p < 0.05; **p < 0.01; ***p < 0.001).

In order to achieve a more comprehensive understanding of individual m6A modification patterns, 983 DEGs associated with the m6A phenotype were identified and shown in the Venn diagram (Supplementary Figure S4G). Gene Ontology (GO) enrichment analysis was performed on the DEGs, and the result demonstrated a significant increment of m6A modification-related and immune-related biological processes in these DEGs, further confirming the crucial role that the m6A modification played in immune regulation in TME (Figure 3F). To further investigate its regulation mechanism, head and neck cancer patients were categorized into three genomic subtypes according to the unsupervised clustering grouping of 983 m6A-related genes, named as m6A gene clusters A, B, and C (Supplementary Figures S5A–D and Figure 4A). This discovery correlated with the three m6A clusters above, further confirming the existence of three distinct m6A modification patterns in head and neck cancer. Similar to the three m6A modification clusters, these three gene cluster phenotypes also present remarkable differences in the expression of m6A regulators (Figure 4C). Gene cluster A exhibited significantly higher expression of eraser genes (ALKBH3, ALKBH5, and FTO) and several reader genes (IGF2BP2, KIAA1429, WTAP, YTHDF1, and YTHDF3), while gene cluster C showed significantly low expression of IGF2BP3, KIAA1429 YTHDF1, YTHDF3, and WTAP, which coordinated with the previous analyses on the three modification patterns. Survival analysis was performed on each gene cluster subsequently (Figure 4B), where gene cluster C showed markedly higher survival advantages. This result further confirmed the accuracy of the above classification of the three phenotypes. Moreover, as shown in the alluvial diagram (Figure 4D), most m6A clusters A and C were classified into gene clusters A and C separately. Almost half of m6A cluster B was grouped into gene cluster B, the majority of the other half classified into gene cluster A, and the rest was categorized into gene cluster C. m6Ascore is a set of a scoring system based on m6A regulatory-related genes functioning to quantify the m6A modification pattern of individual head and neck cancer patients, with the heterogeneity and complexity of individual m6A modification taken into account. It was noticed that a large percentage of gene cluster B and the entire gene cluster C obtained low m6Ascore, while the majority of gene cluster A scored highly. The Kruskal–Wallis test conducted further confirmed the significant difference between m6Ascore of separate gene clusters (Figures 4F,G). m6A cluster A, the immune-excluded phenotype, has the highest m6Ascore among the three clusters, followed by m6A cluster B, which was defined as the immune-desert phenotype. m6A cluster C, the one that fits the description of the immune-inflamed phenotype, scored the lowest. Therefore, it is believed that the m6Ascore is prominently associated with the immune phenotype of head and neck cancer. According to the subsequent one-way ANOVA test, a high m6Ascore was found to be associated with high stroma activity (Figure 4H); thus, a low survival probability was expected. In the subsequent survival analyses, patients with low m6Ascore presented remarkably higher survival probability than the ones with high m6Ascore, reaffirming the close correlation between high m6Ascore with low survival probability (Figures 4B,D). A correlation between low m6Ascore and immune activation-related signature was speculated. Further, Spearman’s analysis was performed to visualize the correlation between m6Ascore and known gene signatures (Figure 4E), aiming to achieve a more in-depth understanding of the characteristics of m6A signature.

**FIGURE 5:**
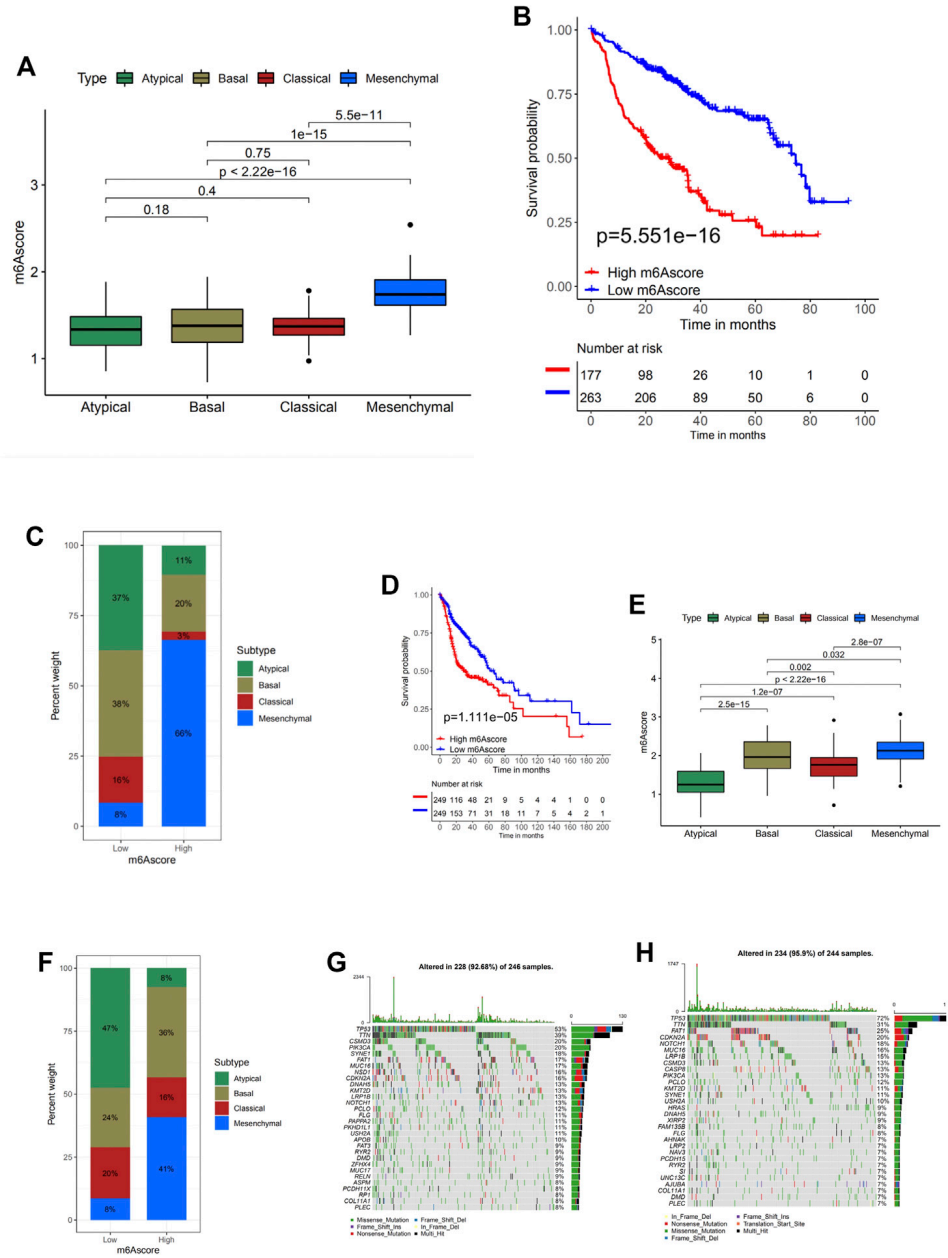
Characteristics of m6A modification in molecular subtypes and tumor somatic mutation. **(A)** Differences in m6Ascore between different subtypes. The Kruskal–Wallis test was used to compare the statistical difference between four subtypes (*p* < 0.0001). **(B)** Survival analyses for low (263 cases) and high (177 cases) m6Ascore patient groups in TCGA-HNSC cohort using Kaplan–Meier curves [hazard ratio (HR), 3.0 (2.12–4.21); *p* < 0.0001, log-rank test]. **(C)** The proportion of molecular subtypes with low and high m6Ascore. Atypical, green; basal subtype, olive; classical subtype, red; mesenchymal subtype, blue. **(D)** Survival analyses for low (249 cases) and high (249 cases) m6Ascore patient groups in the GSE65858 cohort using Kaplan–Meier curves [HR, 1.81 (1.26–2.62); *p* = 0.001, log-rank test]. **(E)** Differences in m6Ascore between different molecular subtypes. The upper and lower ends of the boxes represent the interquartile range of values. The lines in the boxes represent the median value. The Kruskal–Wallis test was used to compare the statistical difference between four molecular subtypes (*p* < 0.0001). **(F)** The proportion of molecular subtypes with low and high m6Ascore. Atypical, green; basal subtype, olive; classical subtype, red; mesenchymal subtype, blue. **(G, H)** The waterfall plot of tumor somatic mutation established by those with high m6Ascore **(G)** and low m6Ascore **(H)**. Each column represents individual patients. The upper barplot shows tumor mutational burden (TMB); the number on the right indicates the mutation frequency in each gene. The right barplot shows the proportion of each variant type.

Next, a multivariate Cox regression model analysis including multiple factors, such as patients’ age, gender, TNM status, progression stage, and m6Ascore, was conducted to examine whether the m6Ascore was capable of serving as an independent prognostic biomarker for head and neck cancer, and the conclusion is positive (Supplementary Figure S6A). Other results obtained from survival analysis in different cohorts (TCGA-HNSC and GSE65858) also indicated that head and neck cancer patients with low m6Ascore presented obvious survival advantages (Figures 5B,D).

Another Kruskal–Wallis test analyzed the correlation between m6Ascore and tumor stages (Supplementary Figure S6B). With a *p*-value = 0.15, no significant difference among head and neck cancer samples at different stages was noticed. Hence, though m6Ascore can predict the progression of the disease to an extent, whether it is also capable of identifying the progression stages is not optimistic.

### Characteristics of m6A Modification in The Cancer Genome Atlas Molecular Subtypes and Tumor Somatic Mutation

The Kruskal–Wallis test conducted on TCGA-HNSC cohort was used to compare the m6Ascore between four molecular subtypes, and the statistical difference was found (Figure 5A). The m6Ascore of different molecular subtypes presented nonnegligible differences, suggesting the potential of utilizing the m6Ascore as an independent biomarker to distinguish different molecular subtypes of head and neck cancer. Subsequent analyses investigating the percent weight of the four molecular subtypes in the high and low m6Ascore groups further confirmed this speculation (Figure 5C). AT and BS subtypes generally have low m6Ascore, while the MC subtype makes up 66% of the high m6Ascore group. To analyze whether a low m6Ascore is remarkably associated with a higher survival rate and clinical advantages, survival analysis was conducted on this cohort (Figure 5B). The answer is positive, verified by a significantly higher survival probability of the low m6Ascore group with a *p*-value of 5.551e−16. In order to verify the results and conclusion above, the same set of analyses was conducted on the GSE65858 cohort, and similar results were found (Figures 5D–F).

Then the differences of somatic mutation distribution between low and high m6Ascore in TCGA-HNSC cohort were analyzed using the maftools package (Figures 5G,H). As the figures suggest, samples with a low m6Ascore presented prominently higher tumor mutational burden (TMB) than samples with high m6Ascore, with the rate of the most significant mutated gene 72% vs. 53%. However, the TMB quantification analyses presented no significant negative correlation between m6Ascore and TMB (Supplementary Figures S7A,B). A possible explanation could be that the tumor somatic mutations are the general pathological changes in head and neck cancer, since the top 10 most significant mutated genes are identical in both the high and low groups.

### m6A Modification Patterns in the Role of Anti-PD-1/L1 Immunotherapy

The stability and prognostic value of the m6Ascore model were further examined by applying the m6Ascore signature established above to independent head and neck cancer cohorts (GSE41613 and GSE42743; Supplementary Figures S8A,B). The results suggested that the m6A modification patterns were relevant with clinical benefit. The predictive advantage was also analyzed with 3-year and 5-year ROC curves (Supplementary Figures S8C,D).

**FIGURE 6:**
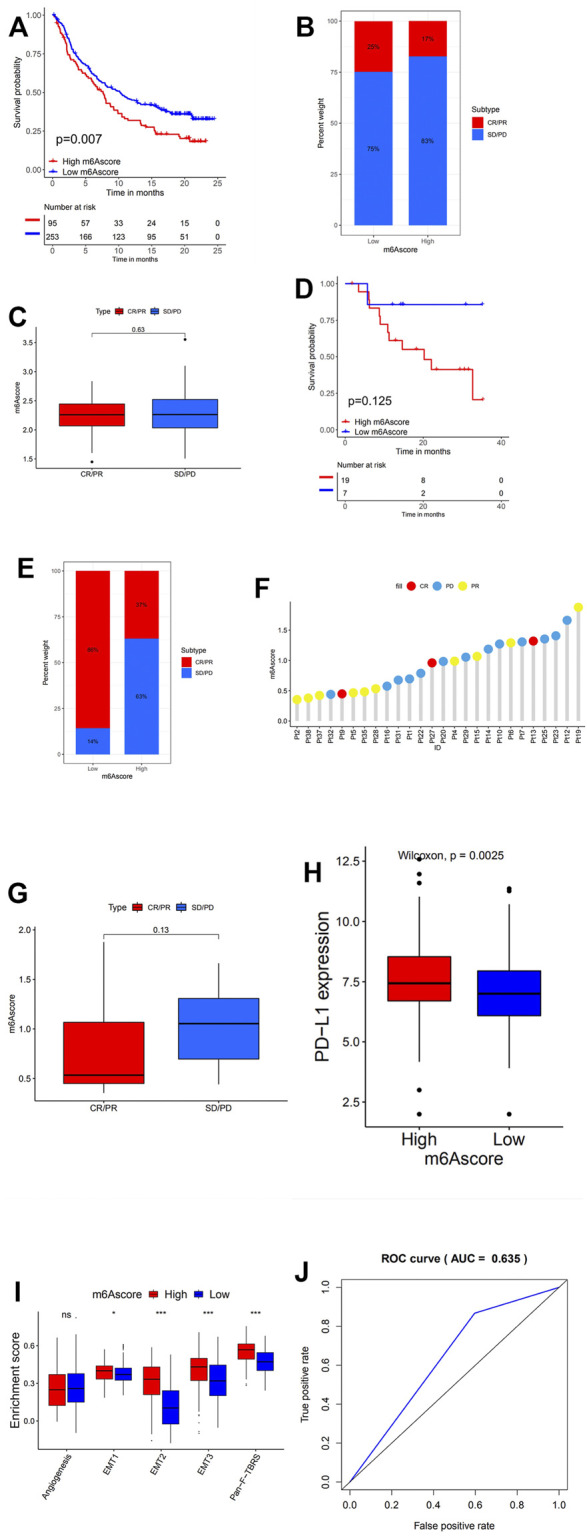
m6A modification patterns in the role of anti-PD-1/L1 immunotherapy. **(A)** Survival analyses for low (253 cases) and high (95 cases) m6Ascore patient groups in the anti-PD-L1 immunotherapy cohort using Kaplan–Meier curves (GSE41613 cohort; hazard ratio (HR), 1.73 (1.20–2.48); *p* = 0.002, log-rank test). **(B)** The proportion of patients with response to PD-L1 blockade immunotherapy in low or high m6Ascore groups. SD, stable disease; PD, progressive disease; CR, complete response; PR, partial response. Responder/Nonresponder: 25%/75% in the low m6Ascore groups and 17%/83% in the high m6Ascore groups. **(C)** Distribution of m6Ascore in distinct anti-PD-L1 clinical response groups. **(D)** Survival analyses for low and high m6Ascore patient groups in the anti-PD1 immunotherapy cohort using Kaplan–Meier curves [GSE42743 cohort; HR, 4.58 (1.23–17.10); *p* = 0.013, log-rank test]. **(E)** The proportion of patients with response to PD-1 blockade immunotherapy in low or high m6Ascore groups. Responder/Nonresponder: 86%/14% in the low m6Ascore groups and 37%/63% in the high m6Ascore groups. **(F)** The correlation of m6Ascore with clinical response to anti-PD-1 immunotherapy. Pt, patients. PD, blue; PR, yellow; CR, red. **(G)** Differences in m6Ascore among distinct anti-PD-1 clinical response groups. **(H)** Differences in PD-L1 expression between low and high m6Ascore groups (*p* < 0.0001, Wilcoxon test). **(I)** Differences in stroma-activated pathways between low m6Ascore and high m6Ascore groups in anti-PD-L1 immunotherapy cohort (*p < 0.05; **p < 0.01; ***p < 0.001). **(J)** The predictive value of the quantification of m6A modification patterns in patients treated with anti-PD-1/L1 immunotherapy (AUC, 0.635).

PD1/PD-L1 blockade immunotherapy has occurred as an unprecedented innovation in the field of cancer therapy. Whether the m6A modification could predict the individual response to ICB therapy was examined in two immunotherapy cohorts. In survival analyses based on the anti-PD-L1 response group, patients with low m6Ascore presented remarkable clinical benefits and prolonged life expectancy (Figure 6A). Although for the anti-PD-1 immunotherapy cohort no survival advantage with statistically significant difference can be found between the low and high m6Ascore groups, we cannot conclude that the m6Ascore is incapable of predicting the prognosis after anti-PD-1 immunotherapy, due to a considerably small sample group (Figure 6D).

In addition, patients with low m6Ascore showed enhanced sensitivity to PD-1 blockade immunotherapy, while the proportion of patients with response to PD-L1 blockade immunotherapy in both high and low m6Ascore was not significant (Figures 6B,E). This was further confirmed through later analyses that demonstrated patients with response to ICB immunotherapy exhibited high m6Ascore (Figures 6C,F). Patients with high m6Ascore also exhibited extensively higher PD-L1 expression, which suggested a potential response to anti-PD-1/L1 immunotherapy (Figure 6H). Stroma in TME was found to be significantly activated in head and neck cancer with high m6Ascore, which indicated high tumor tolerance (Figure 6I).

The above indicated that the qualification of m6A modification patterns as an accurate and robust biomarker for prognosis and clinical response assessment of immunotherapy (Figure 6J).

In conclusion, the conducted tests and analyses elucidated that m6A methylation modification patterns were significantly correlated with tumor immune phenotypes and response to anti-PD-1/L1 immunotherapy, and the construction of the m6A modification scoring system would benefit the classification of individual head and neck cancer patients, the prediction of tumor progression, and the response to anti-PD-1/L1 immunotherapy.

## Discussion

Recently, emerging evidence has indicated that epigenetic regulation, such as m6A regulation of RNA, played a nonnegligible role in innate immune response, inflammation, and antitumor effect through the functioning of m6A regulators. Studies have been conducted focusing on a single type of TME cell or single regulator. However, the correlation between overall TME cell infiltration characterization and the m6A modification patterns remains obscure. Investigating the role of the m6A modification patterns in the characterization of TME infiltration will lead to an in-depth understanding of TME antitumor immune response and provoke new thoughts for better immunotherapy strategies with improved efficacy.

In this study, 21 m6A regulators in head and neck cancer samples were enrolled and analyzed, exhibiting a relatively high frequency of somatic mutations and CNV alteration, which resulted in perturbations on the m6A regulator expression. This suggested a possibility that the m6A modification could be related to the oncogenesis of head and neck cancer. Also, the head and neck cancer samples presented markedly amplificated CNV, which could be completely distinguished from normal samples. The significant genetic heterogeneity and expressional alteration landscape in m6A regulators between normal and head and neck cancer samples demonstrated that the imbalance of m6A regulator expression was closely related to head and neck cancer occurrence and progression. Focusing on the interaction between distinct m6A regulators, cross-talk among the regulators of writers, readers, and erasers may play critical roles in the formation of different m6A modification patterns and TME cell-infiltrating characterization between individual tumors.

Based on the expression of 21 m6A regulators, three modification pattern clusters, termed clusters A, B, and C, were identified and presented with extremely different biological behaviors and TME cell-infiltration characteristics. Cluster A showed prominent enrichment in stromal and carcinogenic activation pathways, and stromal but not parenchymal immune cell infiltration, consistent with the description of the immune-excluded tumor phenotype. Cluster C was characterized by adaptive immune cell infiltration and immune activation, consistent with immune-inflamed tumor phenotype. Cluster B presented significant suppression of immunity, consistent with the immune-desert phenotype. In subsequent survival analyses, the m6A cluster B modification pattern showed prominent survival advantages. The results indicated that the m6A modification patterns had nonnegligible effects on biological behaviors and TME cell-infiltration characterizations of individual tumors.

During the analyses in the GSE65858 cohort to further investigate the characteristics of these m6A modification phenotypes in the different clinical traits and biological behaviors, significant distinction on the m6A transcriptional profile among three separate m6A modification patterns was noticed. Tumors with EMT molecular subtypes were characterized by m6A cluster A methylation modification patterns, exhibiting considerably poor survival probability, while microsatellite instability (MSI) molecular subtypes were characterized by the m6A cluster B modification patterns, presenting better clinical outcomes. The above analyses demonstrated that the m6A cluster A modification pattern was correlated with stromal activation, high malignancy, and rapid progression, while the m6A cluster B modification pattern showed prolonged survival.

Considering the genomic differences that existed among individual m6A regulators, unsupervised clustering was performed based on the m6A signature genes, revealing three distinct m6A modification genomic phenotypes, termed gene clusters A, B, and C. The characteristics of clinical and transcriptome traits in distinct m6A-related genomic phenotypes were markedly different. Immune activation status was frequently observed in gene cluster A, while the status of stromal activation and cancer promotion and almost all patients with EMT subtypes were classified into gene cluster C, further confirming the speculation that m6A methylation modification significantly influences the shaping of different TME landscapes.

However, due to the individual heterogeneity and complexity of the m6A modification in individual patients, a scoring system based on these m6A signature genes termed as m6Ascore quantifying the m6A modification pattern was constructed in addition to the above patient population-based analyses. Subsequent examination elucidated that low m6Ascore was correlated with tumors with gene clusters B and C, m6A cluster C modification pattern, or AT molecular subtypes, exhibiting enhanced stromal activation-related signatures and better clinical outcome. High m6Ascore could be observed in tumors with the m6A cluster A modification pattern, coupled with enhanced immune activation and worse survival outcome. Therefore, m6Ascore can be utilized to better evaluate the m6A modification patterns of individual tumors, achieving a more accurate evaluation of tumors’ TME cell-infiltration characterization. In addition, the potential application of m6Ascore as a prognostic biomarker was also examined and verified, indicating that the prediction was not interfered with by adjuvant CT; low m6Ascore was always associated with an obvious survival advantage in patients both with and without adjuvant CT. Patients in a young age, with diffuse histological subtype or in advanced stages, presented higher m6Ascore and poorer clinical outcomes, suggesting that the m6Ascore could also be utilized to evaluate certain clinical characteristics of patients.

To further investigate the characteristics of the m6A modification in TCGA molecular subtypes and tumor somatic mutation, the m6Ascore was used as a quantifying system. Patients with low m6Ascore had higher TMB and TNB and were usually associated with the mutation of m6A regulators ARID1A and PIK3CA, presenting durable clinical responses to anti-PD-1/L1 immunotherapy and prolonged survival. Subsequent analyses further verified the value of m6Ascore in predicting immunotherapy outcomes, providing an innovative perspective for discovering the interaction between m6A methylation modification and tumor somatic mutations, TME landscape characterization, and response to ICB therapy.

In short, this study has verified the significant role that m6A methylation took in TME cell-infiltration characterization, suggesting a novel approach to quantify individual m6A modification patterns of tumors for further classification, and several potential innovations for future cancer immunotherapy improvement, such as transforming TME cell infiltration characterization through changing m6A modification patterns to benefit the efficacy of ICB immunotherapy, and the development of specified therapeutic strategies for individual immune phenotypes.

## Conclusion

In conclusion, this study elucidated the significant role that m6A methylation modification played in TME immune cell-infiltration characterization. The analyses and evaluation in this work about individual tumor m6A modification patterns will enhance our understanding of TME infiltration characterization and provide innovative thoughts to develop immunotherapy strategies with higher specificity and better efficacy.

## Data Availability

The datasets presented in this study can be found in online repositories. The names of the repository/repositories and accession number(s) can be found in the article/Supplementary Material.
