# Supplementary figures and images for: m6A Regulator-Mediated Methylation Modification Patterns and Tumor Microenvironment Cell-Infiltration Characterization in Head and Neck Cancer
Source: Front Cell Dev Biol. 2022 Feb 7;9:803141. doi: 10.3389/fcell.2021.803141 (PMC8859267; doi:10.3389/fcell.2021.803141)

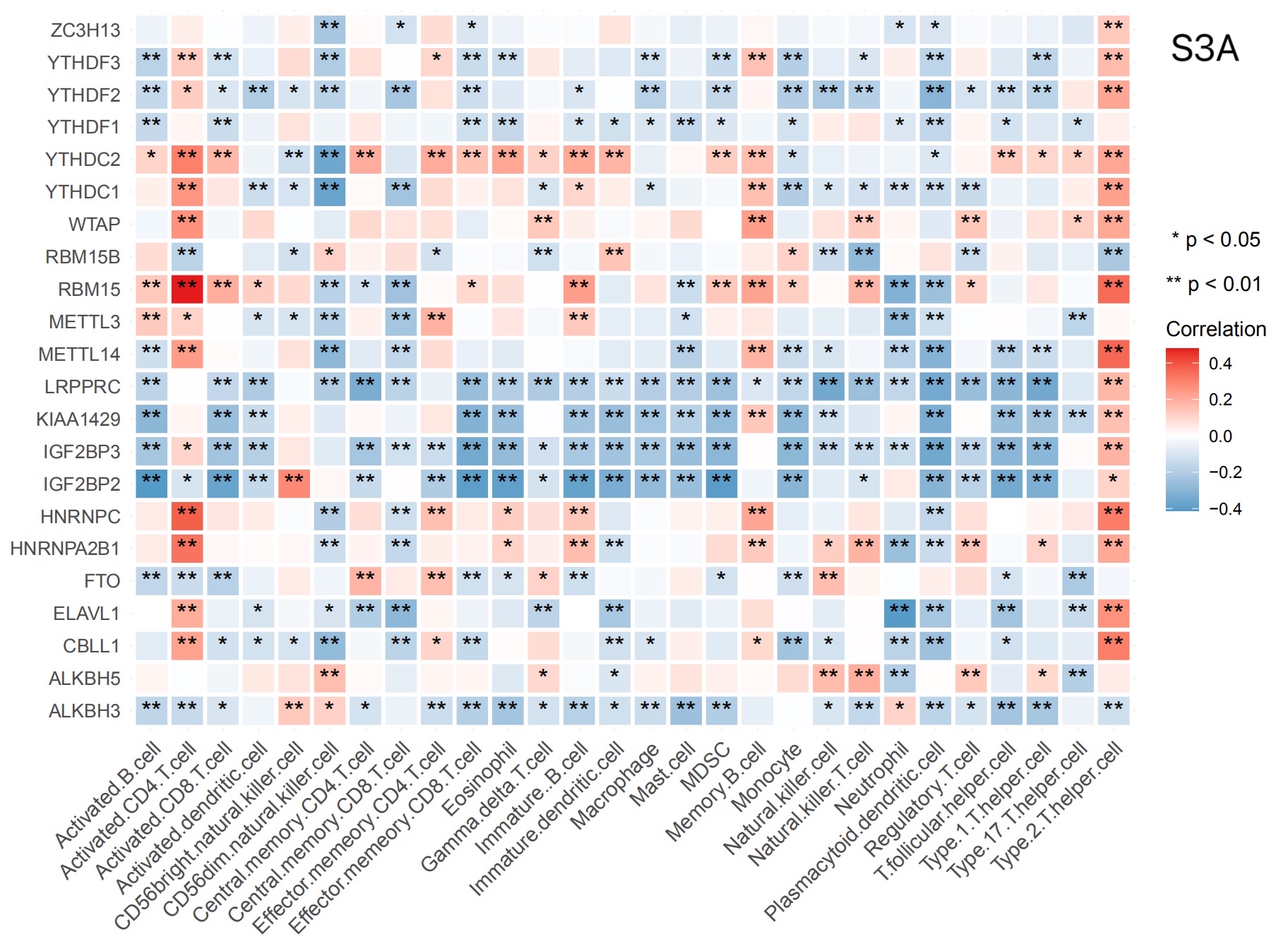

Supplement: Supplementary file 1 [file Image3.JPEG]

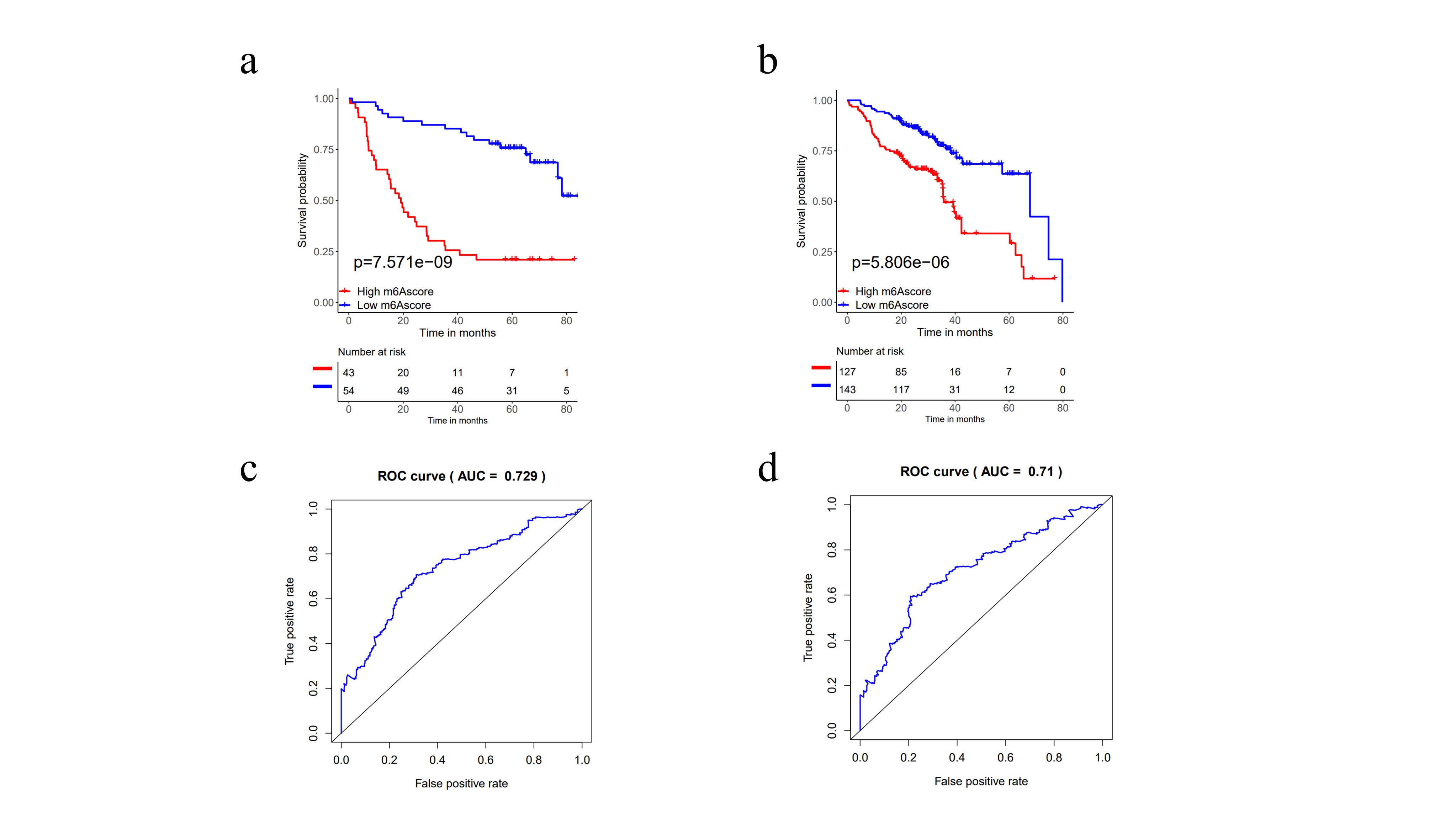

Supplement: Supplementary file 2 [file Image9.TIF]

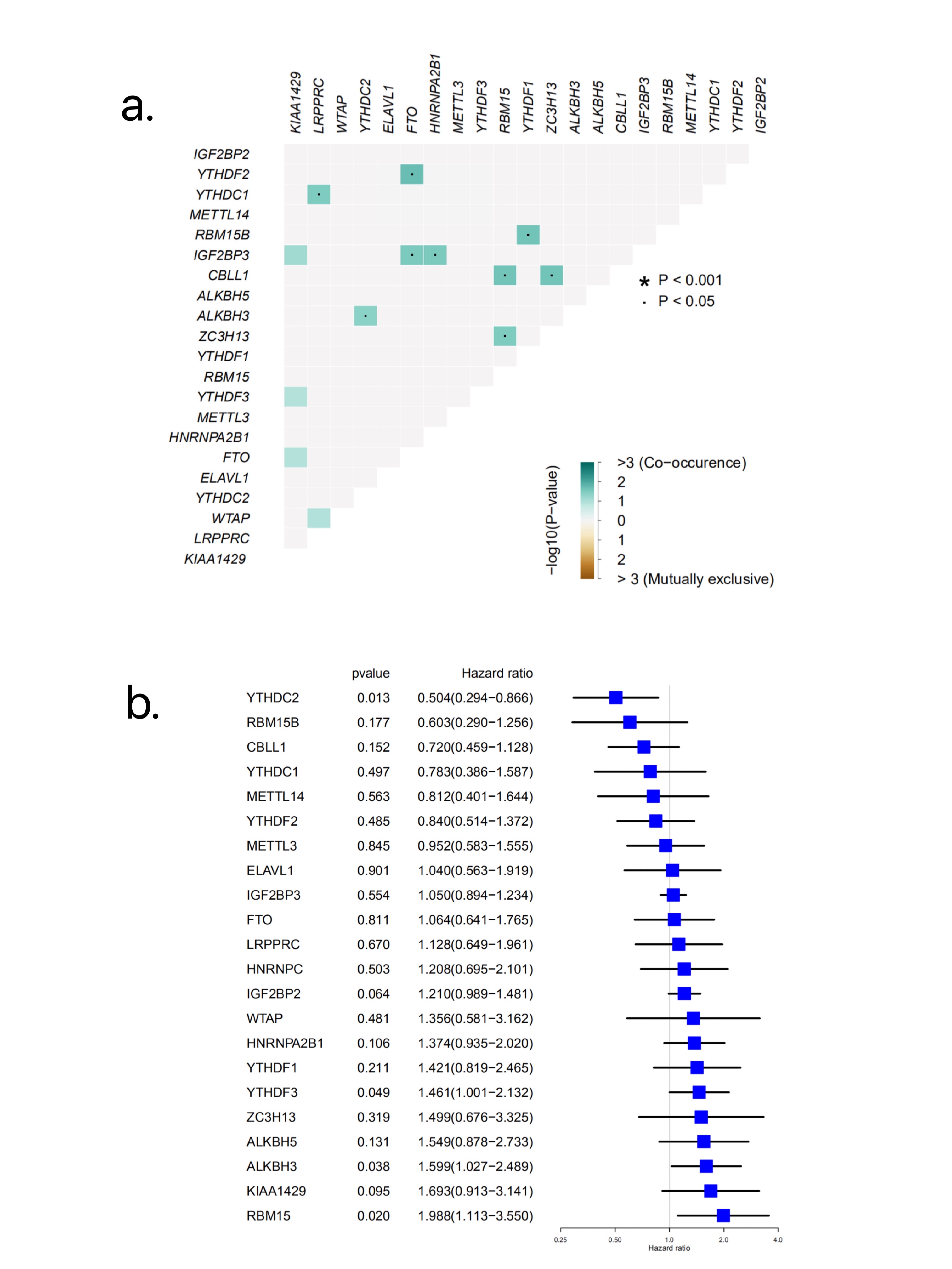

Supplement: Supplementary file 3 [file Image1.JPEG]

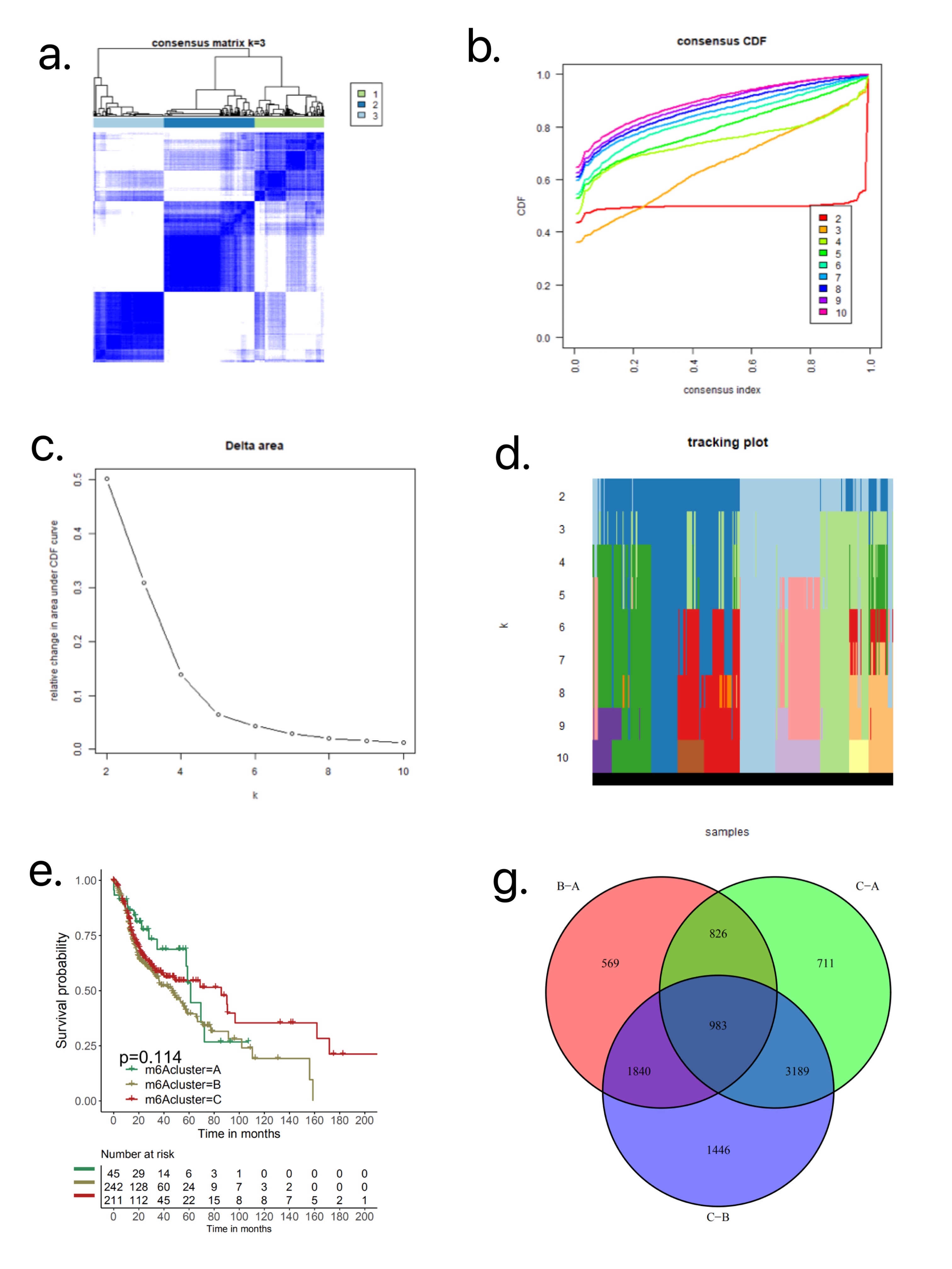

Supplement: Supplementary file 4 [file Image4.JPEG]

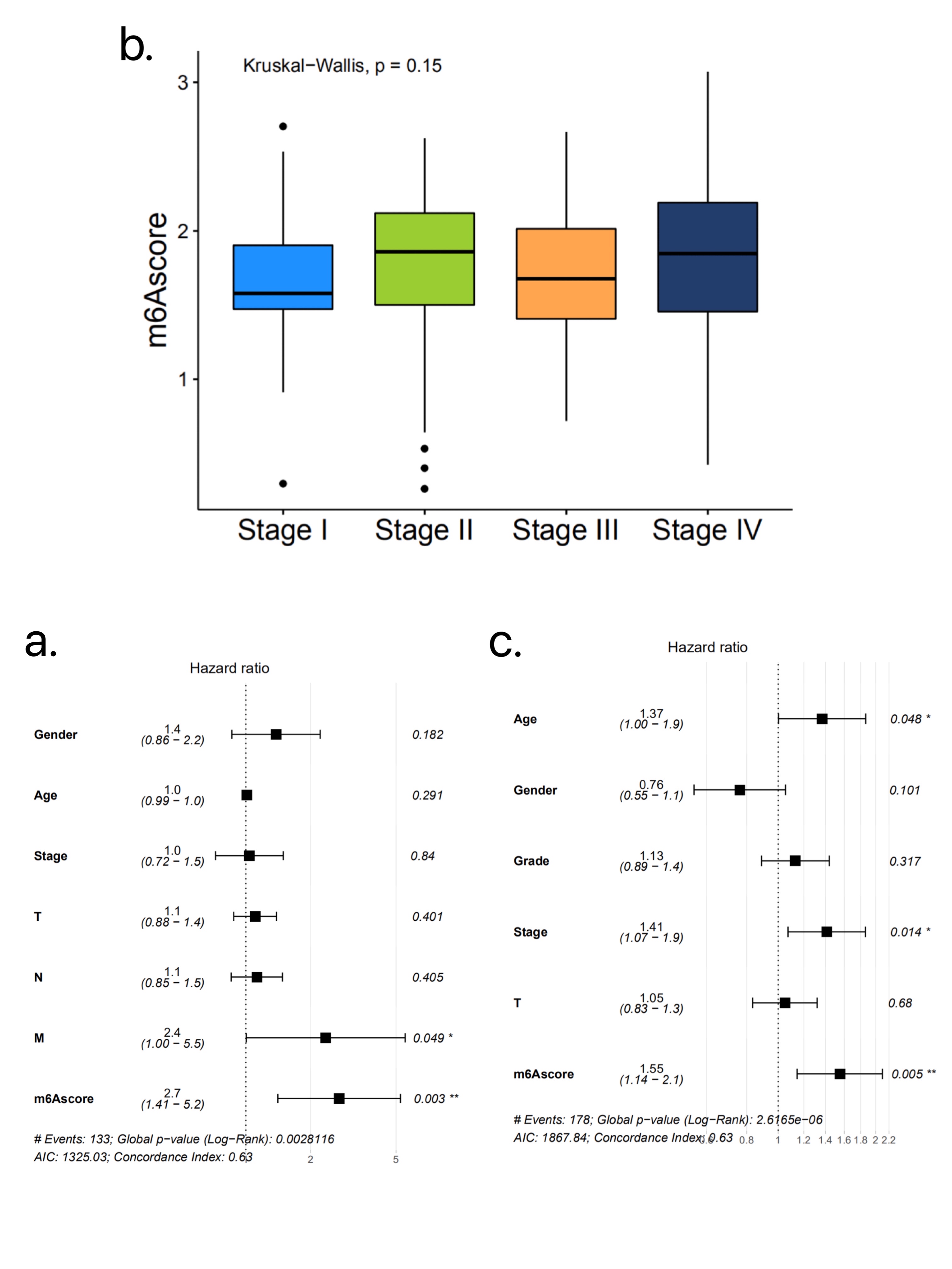

Supplement: Supplementary file 5 [file Image7.JPEG]

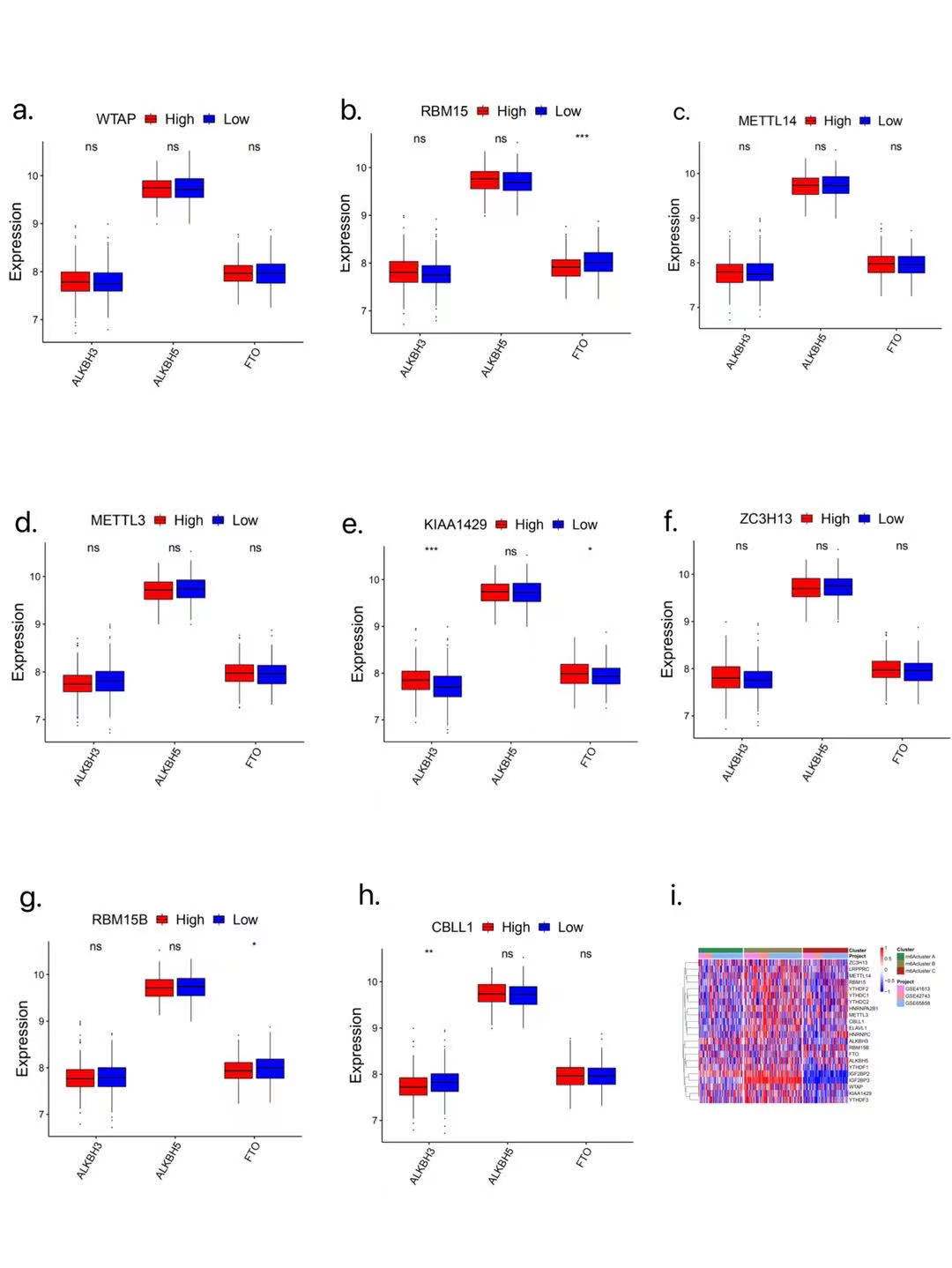

Supplement: Supplementary file 6 [file Image2.JPEG]

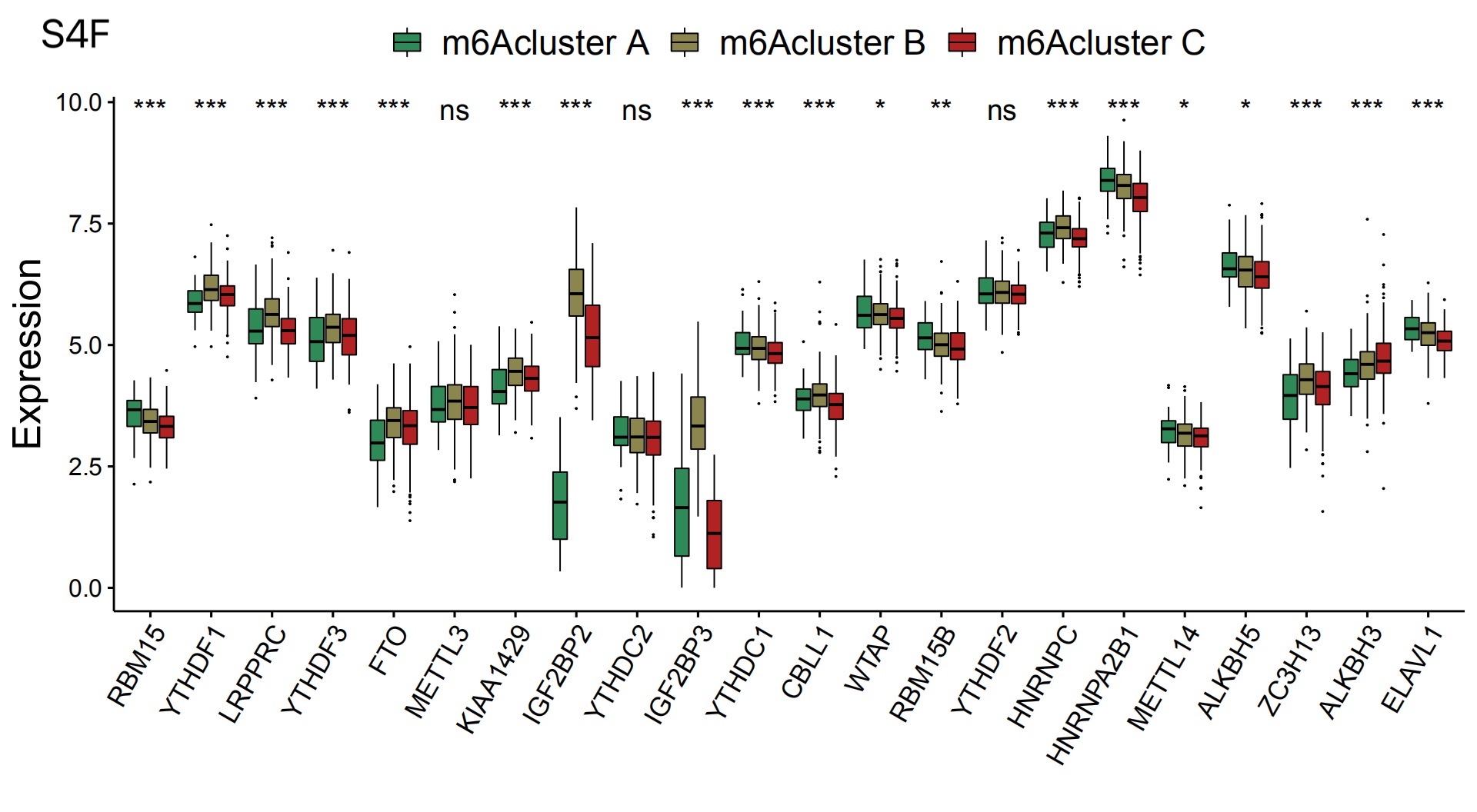

Supplement: Supplementary file 7 [file Image5.JPEG]

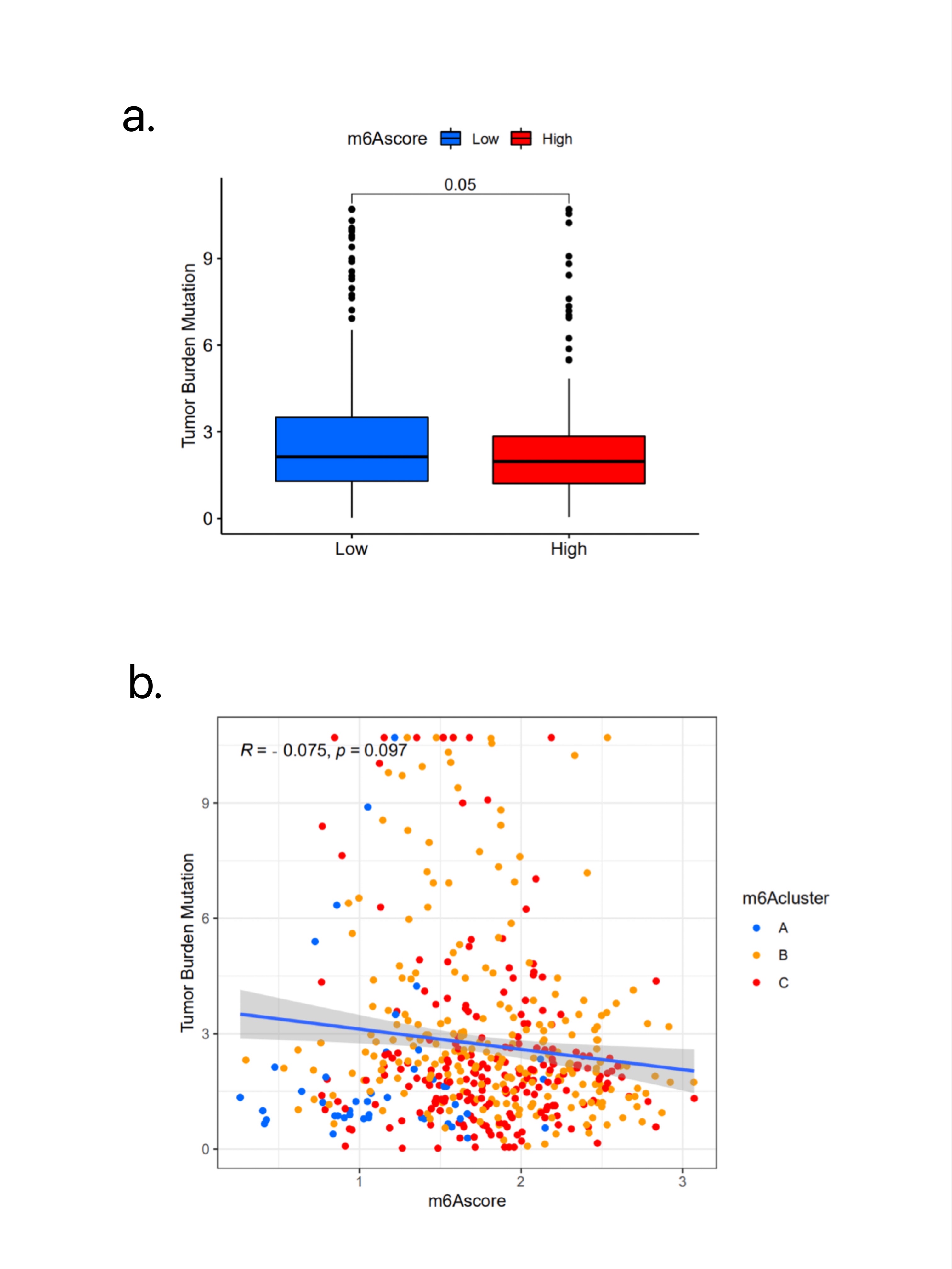

Supplement: Supplementary file 9 [file Image8.JPEG]

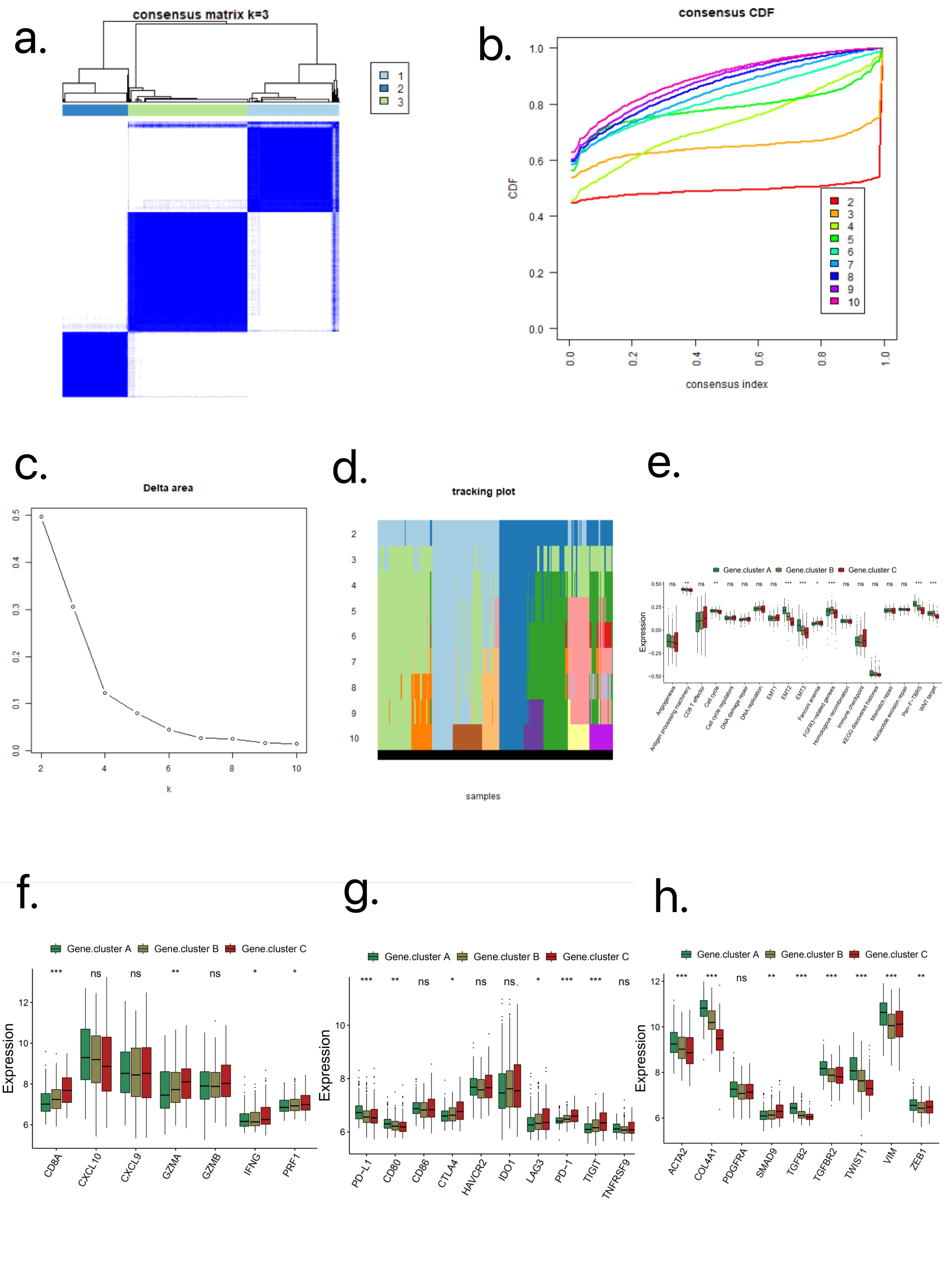

Supplement: Supplementary file 10 [file Image6.JPEG]
